# A randomized placebo-controlled clinical trial of a multi-strain probiotic formulation (Bio-Kult®) in the management of diarrhea-predominant irritable bowel syndrome

**DOI:** 10.1186/s12876-018-0788-9

**Published:** 2018-05-25

**Authors:** Shamsuddin M. Ishaque, S. M. Khosruzzaman, Dewan Saifuddin Ahmed, Mukesh Prasad Sah

**Affiliations:** Department of Gastroenterology, Bangabandhu Sheikh Mujib Medical University, Dhaka, Bangladesh

**Keywords:** BioKult, Diarrhea, Gastrointestinal well-being, IBS, Multi-strain probiotic, Probiotic, Randomized controlled trial, Quality of life

## Abstract

**Background:**

Accumulating evidence supports the view that an imbalance of gut bacteria contributes to IBS, and that increasing the mass of beneficial species may reduce the numbers of pathogenic bacteria and help alleviate symptoms.

**Methods:**

In this double-blind trial 400 adult patients with moderate-to-severe symptomatic diarrhea-predominant IBS (IBS-D) were randomized to treatment with the multi-strain probiotic Bio-Kult® (14 different bacterial strains) or placebo for 16 weeks. The change in severity and frequency of abdominal pain was the primary outcome measure.

**Results:**

Probiotic treatment significantly improved the severity of abdominal pain in patients with IBS-D. A 69% reduction for probiotic versus 47% for placebo (*p* < 0.001) equates to a 145 point reduction on the IBS-severity scoring system (IBS-SSS). The proportion of patients who rated their symptoms as moderate-to-severe was reduced from 100% at baseline to 14% for the multi-strain probiotic at follow-up (month 5) versus 48% for placebo (*p* < 0.001). Also, the number of bowel motions per day from month 2 onwards was significantly reduced in the probiotic group compared with the placebo group (*p* < 0.05). In addition to relieving symptoms, the probiotic markedly improved all dimensions of quality of life in the 34-item IBS-Quality of Life (IBS-QoL) questionnaire. No serious adverse events were reported.

**Conclusions:**

The multi-strain probiotic was associated with significant improvement in symptoms in patients with IBS-D and was well-tolerated. These results suggest that probiotics confer a benefit in IBS-D patients which deserves further investigation.

**Trial registration:**

[Clinicaltrials.gov NCT03251625; retrospectively registered on August 9, 2017].

**Electronic supplementary material:**

The online version of this article (doi:10.1186/s12876-018-0788-9) contains supplementary material, which is available to authorized users.

## Background

Irritable bowel syndrome (IBS) is a common highly prevalent functional gastrointestinal (GI) disorder that places an enormous burden on resource-challenged healthcare systems [1, 2]. It has a heterogeneous clinical phenotype with various symptom combinations including abdominal pain, bloating and altered stool frequency, in the absence of any detectable organic disease with the available clinical tests and examinations [3]. Rome III criteria defines IBS as recurrent abdominal pain or discomfort at least 3 days/month in the last 3 months (symptom onset at least 6 months prior to diagnosis) associated with two or more of the following: improvement with defecation, onset associated with a change in frequency of stool, or onset associated with a change in form (appearance) of stool. The prevalence of IBS varies between geographic regions and populations, and is also dependent upon the diagnostic criteria used [4]. A worldwide prevalence of approximately 10–20% of the adult population has been reported [5] and in the largest analysis to date (41 countries, 288,103 individuals), the mean prevalence among different countries ranged from 1.1% (France and Iran) to 35.5% (Mexico) [1]. Three surveys in Bangladesh have reported IBS prevalence rates of 24.4% (Rome I criteria), 7.7% (Rome II criteria) and 12.9% (Rome III criteria) with a slightly higher rate in female patients [6–8]. Despite being highly symptomatic and negatively impacting the individual’s quality of life (QoL), it has been noted that only about one-third of patients present to their general practitioners [9].

The pathogenesis of IBS is multifactorial, including factors such as genetics, dietary intolerance, alterations in the GI microbiota, small intestinal overgrowth (SIBO), intestinal immune activation, increased intestinal permeability, visceral hypersensitivity, abnormal pain processing, disruption of the gut-brain axis, behavioral pathways and altered GI motility [10–12]. Diagnosis of IBS remains a challenge with no acceptable biochemical, histopathological or radiological tests available. Currently, it is diagnosed using symptom-based criteria initially proposed by Manning which were subsequently modified and incorporated into various iterations of the Rome criteria [13–16]. The role of inflammation and immunological mechanisms in the pathogenesis of IBS symptomatology may be particularly important since, during follow-up, IBS patients tend to have greater mucosal cellularity and other signs of an increased inflammatory response [17–20].

Many drugs have been advocated in the treatment of IBS, including antispasmodics, bulking agents, psychotropic agents, and 5-HT receptor antagonists. However, in the majority of cases these agents have proven to be disappointing for the relief of symptoms, possibly as a result of the heterogeneous pathogenesis of the disease. Probiotics are ‘live microorganisms which when administered in adequate amounts confer a health benefit on the host’ [21]. The rationale for the use of probiotics in the management of IBS is their potential to correct dysbiosis (qualitative and quantitative changes in the microbiota) or to stabilise the host microbiota. A decreased abundance of *Bifidobacterium* and *Lactobacillus* species [22], and an increase in *Gammaproteobacteria* species (a family comprised of numerous pathogens) [23] is frequently reported in IBS studies. Furthermore, PCR-denaturing gradient gel electrophoresis (PCR-DGGE) analysis of fecal samples from IBS patients reveals greater temporal instability of the microbiota compared to healthy controls [24]. The observation of dysbiosis, altered mucosal barrier function, activated immune responses and SIBO all support a potential role for bacteria in both the pathogenesis and treatment of IBS [25–27]. There is a growing body of opinion that an imbalance of gut bacteria contributes to IBS symptoms. Consequently, increasing the mass of beneficial species may help reduce the negative effects of pathogenic bacteria and help alleviate symptoms [28]. The majority of studies to date have been pilot studies [29–34], and this provided the stimulus for the current clinical trial to be undertaken in order to assess whether administration of a multi-strain probiotic (Bio-Kult®; 14 different bacterial strains; 2 billion colony-forming units per capsule) was more effective than placebo at reducing IBS symptoms (especially abdominal pain and frequency) and improving QoL in a large number of patients with diarrhea-predominant IBS (IBS-D) diagnosed in accordance with Rome III criteria.

## Methods

### Study design

The study was a randomized, double-blind, placebo-controlled, equal allocation ratio, parallel-group, clinical trial performed at the BSMMU Gastroenterology department between April 2014 and August 2016. The study was approved by the ethical board of BSMMU, IRB (Institutional Review Board) BSMMU, Dhaka prior to commencement of the study; reference number BSMMU/2015/1011. All participants were informed about the objectives, methodology and purpose of the study in an easily understandable way, and those who agreed to participate were required to provide verbal and written consent prior to entry. The study was conducted in accordance with the ethical principles set out in the Declaration of Helsinki and the ICH Harmonized Tripartite Guideline on Good Clinical Practice. [Clinicaltrials.gov NCT03251625; retrospectively registered August 9, 2017].

### Patients

Male and female patients aged 18 to 55 years with moderate to severe IBS-D diagnosed according to Rome III criteria [recurrent abdominal pain or discomfort (an uncomfortable sensation not described as pain) at least three days a month in the past three months, associated with two or more of the following: improvement with defecation; onset associated with a change in frequency of stool; and onset associated with a change in form (appearance) of stool. The criteria should be fulfilled for the past three months with symptom onset at least six months before diagnosis]. Patients classified as IBS-D (diarrhea predominant) had > 25% loose/wet motions (Bristol stool scale 6–7) and < 25% firm/hard motions (Bristol stool scale 1–2). The severity of IBS was determined by the IBS Severity Scoring System (IBS-SSS) as described below.

The following alarm features were required to be absent as part of screening to minimize the risk of missing important organic diseases; rectal bleeding, anemia, unexplained weight loss, nocturnal diarrhea, and a family history of organic GI diseases (e.g. colon cancer or inflammatory bowel disease). All patients agreed not to start any other drug treatment unless clinically indicated. Exclusion criteria included: treatment with probiotics within last three months; concurrent severe illness (cancer, uncontrolled diabetes mellitus, hepatic, renal or cardiac dysfunction, and hyper- or hypothyroidism); previous GI surgery; chronic organic bowel disorders (e.g. inflammatory bowel disease, tuberculosis, diverticular disease, etc); treatment with antibiotics in the two months prior to enrolment; pregnancy or lactation. To exclude other diagnoses, patients fulfilling the inclusion criteria for IBS were screened using the following tests: full blood count (FBC), erythrocyte sedimentation rate (ESR), C-reactive protein (CRP), antibody testing for coeliac disease (endomysial antibodies or tissue transglutaminase).

### Procedures

**Fig. 1:**
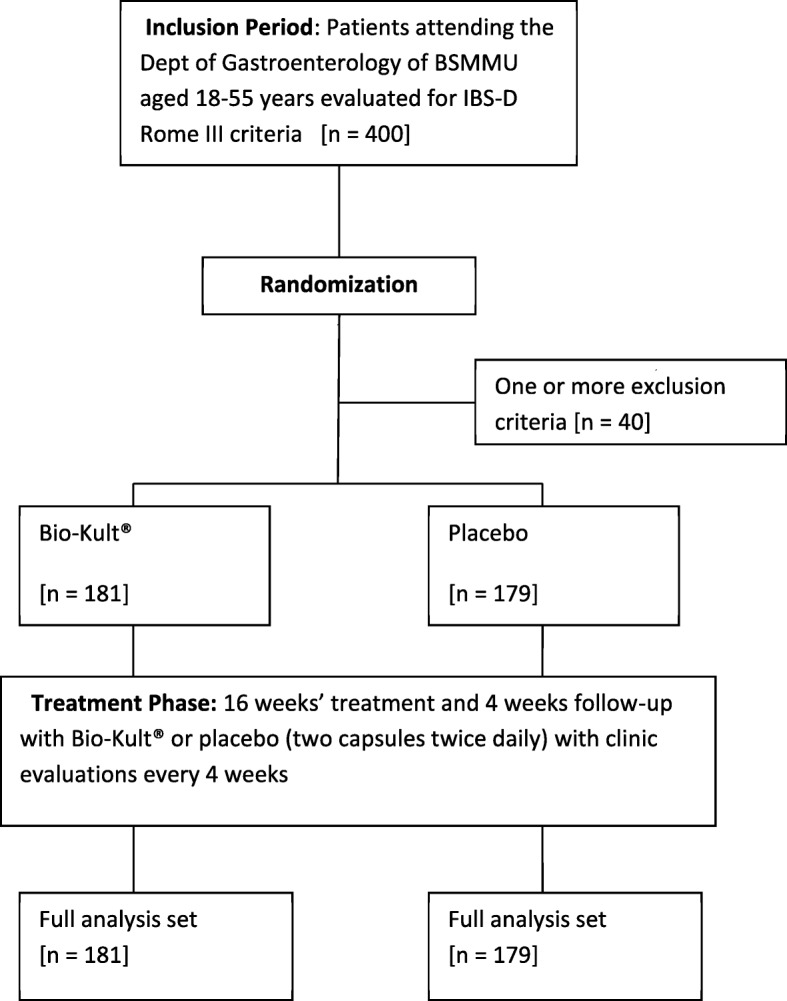
Study protocol

Patients fulfilling the inclusion criteria in the absence of exclusion criteria or an alternative diagnosis, and who provided a written informed consent form, were included in the study and comprised the randomization group (Fig. 1). All participants were advised to maintain their usual dietary practices throughout the study.

The aim was to recruit approximately 400 IBS-D patients (see Statistical Analyses section). Randomization into two groups (equal ratios) was performed by an independent statistician using the randomizer software (www.randomizer.org). One group received the multi-strain probiotic Bio-Kult® (Probiotics International Ltd. (Protexin), Somerset, UK), two capsules twice daily (manufacturer-recommended daily dosage), while the other group received identical placebo capsules (the filler was microcrystalline cellulose in a vegetable capsule made of hydroxypropyl methylcellulose), two capsules twice daily. Bio-Kult® is a capsule formulation containing 14 different bacterial strains (2 billion CFUs per capsule) and a dosage of two capsules twice a day is equivalent to 8 billion CFUs / day. The 14 different bacterial strains in Bio-Kult® are: *Bacillus subtilis* PXN 21, *Bifidobacterium* spp. (*B. bifidum* PXN 23, *B. breve* PXN 25, *B. infantis* PXN 27, *B. longum* PXN 30), *Lactobacillus* spp. (*L. acidophilus* PXN 35, *L. delbrueckii* spp. Bulgaricus PXN39, *L. casei* PXN 37, *L. plantarum* PXN 47, *L. rhamnosus* PXN 54*, L.helveticus* PXN 45, *L. salivarius* PXN 57), *Lactococcus lactis* PXN 63, and *Streptococcus thermophilus* PXN 66]. The capsules were administered before or during a meal for a total of 16 weeks; patients were followed-up one month after this time.

An independent data monitor maintained treatment codes and allocation, which was locked until all analyses had been completed. Thus, the clinical trial was performed double-blind with all patients and clinical staff unaware of which treatment had been allocated.

Before starting treatment each individual underwent a baseline assessment during which demographic data, IBS symptoms and QoL data were recorded. During treatment patients were required to return to the clinic once a month for reassessment of IBS symptoms and QoL, and to report any adverse events (AE) that had occurred.

The IBS-Severity Scoring System (IBS-SSS) questionnaire was completed at baseline, at each monthly clinic visit and after one month’s follow-up. The IBS-SSS is a 5-item instrument used to measure severity of abdominal pain, frequency of abdominal pain (number of days with abdominal pain over the last 10 days), severity of abdominal distension, dissatisfaction with bowel habits, and interference with quality of life, each on a 100-point scale [35]. The items are summed and thus the total score can range from 0 to 500 points. IBS severity has the following defined ranges: mild 75–174, moderate 175–300, and severe > 300. The IBS-SSS was completed by the physician at each clinic visit.

The IBS-QoL questionnaire is a 34-item measure constructed specifically to assess QoL impairment due to IBS symptoms [36]. Each item is scored on a five-point scale (1 = not at all, 5 = a great deal) that represents one of eight dimensions (dysphoria, interference with activity, body image, health-related worries, food avoidance, social reactions, sexual dysfunction, and relationships). Items are scored to derive an overall total score of IBS related QoL. To facilitate score interpretation, the summed total score is transformed to a 0–100 scale ranging from zero (poor QoL) to 100 (maximum QoL). The QoL instrument was translated into Bengali and given to each patient before treatment was started; the patient completed the form each month and all data were recorded and entered onto a data sheet.

### Efficacy assessments and endpoints

The aim of this study was to determine whether administration of a multi-strain probiotic was more effective than placebo at reducing GI symptoms and improving QoL in patients with moderate to severe IBS-D.

#### Primary endpoint

- The change in severity and frequency of abdominal pain on the IBS-SSS during treatment with a multi-strain probiotic or placebo, and compared with baseline.

#### Secondary endpoints

- The change in other GI symptom severity scores (including stool consistency, frequency, and bloating) on the IBS-SSS during treatment with a multi-strain probiotic and placebo, and compared with baseline.
- The change in QoL parameters (using a validated IBS-QoL questionnaire) during treatment with a multi-strain probiotic and placebo, and compared with baseline.
- To assess any AEs reported during treatment with a multi-strain probiotic and placebo.

### Sample size

The sample size for this trial was determined using the formula:

$$ {\mathrm{n}}_1=2{\sigma}^2{\left({\mathrm{Z}}_{\beta }+\mathrm{Z}\alpha \right)}^2/{\left({\mu}_1\hbox{--} {\mu}_2\right)}^2 $$

Where:

$$ {\mathrm{Z}}_{\beta }=0.84\ \mathrm{a}\mathrm{t}\ 80\%\mathrm{power};\mathrm{and}\ \mathrm{Z}\alpha =1.96\ \mathrm{a}\mathrm{t}\ \mathrm{a}\ 95\%\mathrm{confidence}\ \mathrm{interval}. $$

μ_1_ – μ_2_ represents the minimum clinically important difference which was set at 30% (this is advocated as the minimal clinically significant reduction for probiotics). A standard deviation (σ) of 87.77 for IBS-SSS from a similarly designed study was reported by Sisson and colleagues and was used in the sample size calculation [29]. Based on these assumptions the sample size was calculated to be 135 per treatment group (270 in total). Allowing for dropouts and non-adherence we estimated that the sample size should be increased to a total of approximately 384 patients (192 per group). In practice the first 400 patients with IBS-D attending the BSMMU Gastroenterology department between April 2014 and August 2016 and who provided written informed consent entered the study and were randomized to treatment.

### Statistical analyses

All analyses were performed in the per-protocol (PP) set, i.e. treated patients that had no major protocol violations, met the minimum protocol requirements, and who were able to be evaluated for the primary endpoint. IBS-SSS symptom scores and IBS-QoL instrument scores were expressed as means ± standard deviation (SD) and analysed using the Student’s unpaired t-test. Categorical data are presented as frequencies/percentages and were analysed using the chi-square test. The relationship between different variables was investigated using Pearson’s correlation coefficient. *P* values < 0.05 were considered to be statistically significant. All analyses were performed using a computer based SPSS program (version 13.0).

## Results

A total of 400 patients with IBS-D diagnosed by Rome III criteria and who met the inclusion criteria, were randomized to 16 weeks’ treatment. A total of 360 patients completed the study and comprised the PP analysis set. Of these, 181 patients received multi-strain probiotic treatment and 179 received placebo (Fig. 1).

### Demographics and baseline characteristics

The treatment groups were comparable with respect to mean (±SD) age (32.2 ± 10.1 and 31.7 ± 9.7 years for probiotic and placebo,respectively) and gender (male / female ratio 3.7/1 with a slightly higher rate in the placebo group: 4.3/1 vs. 3.2/1 in the multi-strain probiotic group (*p* = 0.179) (Table 1). The two groups were comparable for the proportion of patients with moderate/severe IBS-D: 21.5/78.5% in the probiotic group and 29.1/70.9% in the placebo group (*p* = 0.101).

**Table 1:** Patient demographics

Variable	Probiotic (*n* = 181)	Placebo (*n* = 179)	*P*-value
Age (years) [mean ± SD]	32.2 ± 10.1	31.7 ± 9.7	0.642
Gender (males/ females)	136/45	145/34	0.179
IBS-D (Rome III criteria)	181 (100)	179 (100)	NS
Moderate	39 (21.5)	52 (29.1)	0.101
Severe	142 (78.5)	127 (70.9)	
Occupation:			
Service industry	59	50	0.043
Student	51	38	
Business person	19	23	
Housewife	22	25	
Worker (painter, tailor, driver, farmer)	30	43	

### Changes in IBS symptom scores

**Fig. 2:**
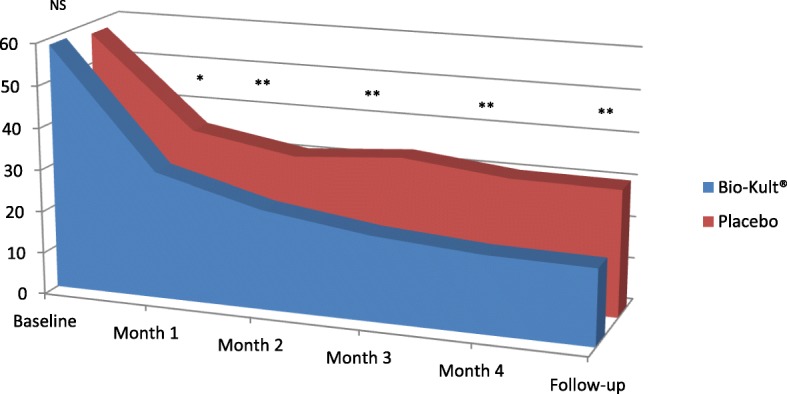
IBS-SSS abdominal pain ratings with probiotic (Bio-Kult®) or placebo (16 weeks’ treatment and 1-month follow-up). The lower the score the less the pain: * *p* = 0.002; ** *p* < 0.001; NS = not significant

Results pertaining to changes in IBS symptom scores are presented in Table 2. For the 5-item IBS-SSS endpoints (abdominal pain, frequency of abdominal pain (number of days with abdominal pain over the last 10 days), severity of abdominal distension, dissatisfaction with bowel habits, and interference with life), as well as the overall IBS-SSS score, the differences between the multi-strain probiotic and placebo groups were statistically significant at all time points. In the probiotic group the overall IBS-SSS was reduced by 145 points within 30 days (117 points in the placebo group) and by 223 points by month 5 (157 points in the placebo group). This is highlighted by a highly significant reduction in abdominal pain levels (primary outcome measure) during four months’ treatment and at follow-up (Fig. 2). At follow-up the abdominal pain level had decreased by 69% (decrease of 40 points) in the multi-strain probiotic group versus 47% (decrease of 27 points) in the placebo group (58.5 ± 11.1 to 18.1 ± 15.2 vs. 57.2 ± 10.6 to 30.2 ± 19.9; *p* < 0.001).

**Table 2:** IBS symptom scores at baseline, during 16 weeks’ treatment and after one month’s follow-up

	Probiotic (Bio-Kult®) (n = 181)	Placebo (n = 179)	*P*-value
Overall IBS-SSS scores			
Before treatment	333.0 ± 40.4	332.9 ± 42.0	0.992
Month 1	187.9 ± 61.3	215.4 ± 75.0	< 0.001
Month 2	146.5 ± 76.4	188.0 ± 92.0	< 0.001
Month 3	122.0 ± 78.3	199.5 ± 104.1	< 0.001
Month 4	115.2 ± 75.0	179.7 ± 100.2	< 0.001
Month 5	110.0 ± 71.8	176.0 ± 100.0	< 0.001
IBS-SSS: Severity score of abdominal pain			
Before treatment	58.5 ± 11.1	57.2 ± 10.6	0.264
Month 1	30.3 ± 14.8	35.3 ± 15.9	0.002
Month 2	23.8 ± 16.2	31.1 ± 18.8	< 0.001
Month 3	20.3 ± 15.8	33.1 ± 19.7	< 0.001
Month 4	18.5 ± 16.2	30.4 ± 20.3	< 0.001
Month 5	18.1 ± 15.2	30.2 ± 19.9	< 0.001
IBS-SSS: Number of days in the last 10 days with pain			
Before treatment	7.7 ± 2.3	8.1 ± 2.3	0.056
Month 1	3.6 ± 2.1	4.4 ± 2.5	0.001
Month 2	2.9 ± 2.3	3.8 ± 2.7	0.001
Month 3	2.5 ± 2.2	4.2 ± 2.8	< 0.001
Month 4	2.4 ± 2.1	4.1 ± 3.2	< 0.001
Month 5	2.2 ± 1.9	3.9 ± 3.0	< 0.001
IBS-SSS: Severity score of abdominal distension			
Before treatment	58.5 ± 11.5	58.9 ± 12.0	0.695
Month 1	34.2 ± 16.2	38.4 ± 19.3	0.028
Month 2	25.7 ± 16.9	35.6 ± 20.2	< 0.001
Month 3	21.1 ± 16.4	37.5 ± 22.3	< 0.001
Month 4	20.1 ± 16.6	36.3 ± 23.3	< 0.001
Month 5	19.6 ± 15.8	35.9 ± 23.5	< 0.001
IBS-SSS: Satisfaction score for bowel symptoms			
Before treatment	71.0 ± 9.7	69.6 ± 13.1	0.256
Month 1	44.9 ± 14.2	50.3 ± 16.9	0.001
Month 2	34.3 ± 17.2	43.0 ± 19.6	< 0.001
Month 3	29.6 ± 18.3	45.5 ± 22.5	< 0.001
Month 4	28.0 ± 18.9	43.3 ± 23.8	< 0.001
Month 5	26.5 ± 19.1	42.2 ± 25.0	< 0.001
Highest number of bowel motions per day			
Before treatment	6.1 ± 2.6	5.6 ± 1.8	0.024
Month 1	3.3 ± 1.4	3.3 ± 1.4	0.891
Month 2	2.9 ± 1.2	3.2 ± 1.3	0.043
Month 3	3.0 ± 1.5	3.6 ± 1.3	< 0.001
Month 4	2.7 ± 1.5	3.5 ± 1.3	< 0.001
Month 5	2.5 ± 1.4	3.4 ± 1.4	< 0.001
Passing excess mucus [no. pts. (%)]			
Before treatment	181 (100.0)	179 (100.0)	
Month 1	170 (93.9)	177 (98.9)	0.012
Month 2	175 (96.7)	175 (97.8)	0.748
Month 3	175 (98.9)	177 (98.9)	0.157
Month 4	171 (94.5)	174 (97.2)	0.195
Month 5	168 (92.8)	171 (95.5)	0.272
IBS-SSS: Score of IBS affecting or interfering with life			
Before treatment	68.6 ± 12.6	66.1 ± 11.1	0.049
Month 1	42.5 ± 15.0	47.4 ± 16.6	0.004
Month 2	33.4 ± 17.2	40.6 ± 19.7	< 0.001
Month 3	26.0 ± 17.9	41.8 ± 23.4	< 0.001
Month 4	24.9 ± 16.5	29.1 ± 19.6	0.028
Month 5	23.4 ± 17.4	28.2 ± 20.1	0.015

The unpaired t-test was used to determine the level of statistical significance

In addition to improvements in IBS-SSS ratings, the number of bowel motions/day was significantly reduced from month 2 onwards in the multi-strain probiotic group compared with the placebo group (Table 2). In contrast the passing of excess mucus was similar in the two treatment groups.

At baseline all patients rated their symptoms as moderate to severe (Table 3). However, at the end of the follow-up period 52.5% of patients in the multi-strain probiotic group rated their symptoms as mild compared with 39.1% in the placebo group (*p* < 0.001). Moreover, the number of patients symptom free at the end of the study was 33.7% in the multi-strain probiotic group compared with only 12.8% in the placebo group (p < 0.001).

**Table 3:** Severity of symptoms at baseline, during 16 weeks’ treatment and after one month’s follow-up)

Severity of IBS-D	Probiotic (Bio-Kult®) (n = 181)	Placebo (n = 179)	*P*-value
Baseline			
Moderate	39 (21.5)	52 (29.1)	0.101
Severe	142 (78.5)	127 (70.9)	
Month 1			
Symptoms free period	2 (1.1)	2 (1.1)	0.086
Mild	78 (43.1)	58 (32.4)	
Moderate	91 (50.3)	99 (55.3)	
Severe	10 (5.5)	20 (11.2)	
Month 2			
Symptoms free period	16 (8.8)	18 (10.1)	< 0.001
Mild	112 (61.9)	61 (34.1)	
Moderate	42 (23.2)	82 (45.8)	
Severe	11 (6.1)	18 (10.1)	
Month 3			
Symptoms free period	54 (29.8)	20 (11.2)	< 0.001
Mild	98 (54.1)	62 (34.6)	
Moderate	23 (12.7)	57 (31.8)	
Severe	6 (3.3)	40 (22.3)	
Month 4			
Symptoms free period	56 (30.4)	22 (11.2)	< 0.001
Mild	99 (54.7)	68 (38.0)	
Moderate	21 (11.6)	66 (36.9)	
Severe	6 (3.3)	23 (12.8)	
Follow-up: Month 5			
Symptoms free period	61 (33.7)	23 (12.8)	< 0.001
Mild	95 (52.5)	70 (39.1)	
Moderate	21 (11.6)	65 (36.3)	
Severe	4 (2.2)	21 (11.7)	

The unpaired Chi-square test was used to determine the level of statistical significance

Comparison of IBS symptom score improvements by age and gender in the multi-strain probiotic group revealed no statistically significant differences by month 5. In contrast, in the placebo group, patients aged 30 years and over had significantly improved pain (p < 0.001) and abdominal bloating (*p* = 0.041) scores compared with patient aged < 30 years.

### Changes in IBS-QoL scores

**Fig. 3:**
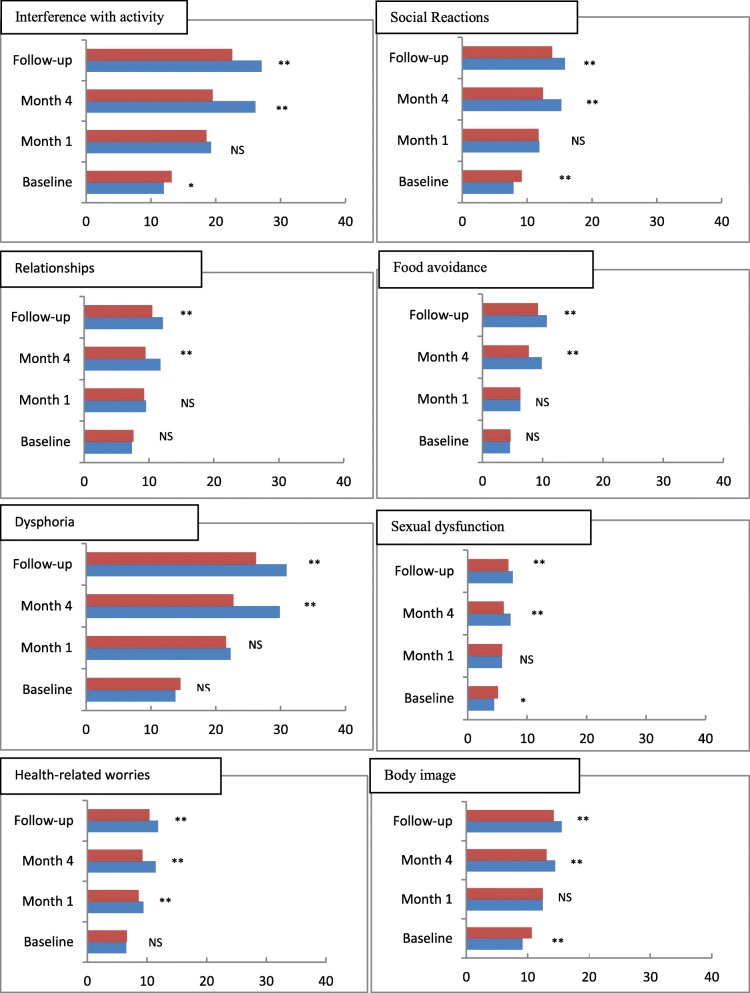
Individual dimension scores for IBS-QoL during 16 weeks’ treatment with multi-strain probiotic (Bio-Kult®; blue square) or placebo (red square). * *p* < 0.05; ** p < 0.001; NS, not significant. Note: In this scoring system, higher scores indicate better QoL

Changes in overall QoL as assessed by the IBS-QoL questionnaire are shown in Table 4. From month 2 onwards there was a statistically significant improvement in QoL in the multi-strain probiotic group compared with the placebo group. Figure 3 details the results for the eight individual dimensions on the IBS QoL questionnaire. Food avoidance, sexual dysfunction and health-related worries had the most negative impact on QoL at baseline in both groups of patients. The benefits of multi-strain probiotic therapy were consistent across all eight dimensions with a steady increase over time. After 4 months’ treatment, and at follow-up, the ratings in the probiotic group were statistically significantly higher than in the placebo group (*p* < 0.001 in all cases). Neither age nor gender had any statistically significant effects on IBS-QoL scores at month 5.

**Table 4:** IBS-QoL scores at baseline, during 16 weeks’ treatment and after one month’s follow-up)

	Probiotic (Bio-Kult®) (n = 181)	Placebo (n = 179)	*P*-value
Before treatment	22.6 ± 10.5	27.5 ± 13.0	< 0.001
At 1st month	46.5 ± 13.6	44.8 ± 15.8	0.270
At 2nd month	59.0 ± 18.9	48.7 ± 20.3	< 0.001
At 3rd month	66.4 ± 21.6	47.6 ± 22.9	< 0.001
At 4th month	68.3 ± 21.8	48.4 ± 24.5	< 0.001
At 5th month	72.0 ± 16.5	58.5 ± 16.8	< 0.001

The unpaired t-test was used to determine the level of statistical significance

*Note*: In this scoring system, higher scores indicate better QoL

### Tolerability and safety

Both multi-strain probiotic treatment and placebo were well-tolerated with no treatment-related adverse events (AEs) reported during the study.

## Discussion

### Summary of main findings

According to Rome III criteria, abdominal pain or discomfort are hallmark determinants for the initial diagnosis of IBS and changes in bowel movements define different subtypes [16]. Typically, the pain or discomfort is related to defecation, or its onset is associated with an increase or decrease in the frequency or form of stool, which can be further exacerbated by stressful life events. Therefore, changes in the severity of abdominal pain are a reliable measure of treatment outcome and were assessed in this cohort using IBS-SSS questionnaire, which incorporates two pain-related items [29, 35, 37]. A decrease of > 50 points in the IBS-SSS score is indicative of a clinical improvement [35], although it has been suggested that a minimum reduction of 95 points is needed to show a clinically relevant change in symptoms [37]. In the current study an average reduction in the overall IBS-SSS score was 145 points achieved within 30 days of intervention and this was increased to over 200 points after 16 weeks of treatment. These reductions with multi-strain probiotic are clinically meaningful and they were not significantly affected by age or gender. Furthermore, they are markedly greater than changes in IBS-SSS reported previously with a single strain probiotic after 12 weeks’ treatment (reduction of 50 points after 12 weeks) [38] and in multi-strain probiotic trial (reduction of 63 points after 12 weeks) [29]. With respect to the primary outcome, 16 weeks supplementation with a multi-strain probiotic, reduced the abdominal pain level by 69% versus 47% in the placebo group (58 points to 18 points vs. 57 points to 30 points; *p* < 0.001). Over 85% of patients in the probiotic group reported an improvement in their severity category, whereas nearly half (48%) of the placebo group did not see an improvement in their severity category. The relatively high response in the placebo group has been previously reported in functional bowel disorder studies, which was shown to have a negative correlation with study duration [30]. As per European Medicines Agency “Guidance on The Evaluation of Medicinal Products for the Treatment of IBS”, a responder has to demonstrate abdominal pain score which has improved at least 30% compared to baseline [39]. In the current study key symptoms of IBS were statistically significantly improved compared with placebo, with the change in most parameters exceeding 30%.

There is accumulating evidence showing that certain probiotics may be capable of significantly reducing abdominal pain, abdominal distension and flatulence while, at the same time, increasing health-related QoL in IBS patients [40]. Likewise in the current study, in addition to relieving symptoms, multi-strain probiotic treatment was shown to markedly improve all dimensions of QoL evaluated using the 34-item IBS-QoL questionnaire. Beneficial changes were noted within one month of starting treatment and, after 4 months’ treatment, the improvements were statistically significant (*p* < 0.001) for all eight measures included in this QoL instrument. During the course of this clinical trial, multi-strain probiotic treatment and placebo were equally well-tolerated with no treatment-related AEs reported.

### Comparison with existing literature

A recently published systematic review of probiotics undertaken by the British Dietetic Association (BDA) found that of 35 probiotic RCTs analysed, almost three-quarters were potentially pilot studies with a sample size too small (< 50 patients/group) to develop any probiotic-specific clinical recommendations [41]. The sample size (*N* = 360) of the current study was based on robust statistical analyses to achieve adequate power and represents one of the largest probiotic IBS clinical trials to date. Moreover, the research recommendations from the BDA review called for high-quality RCTs of probiotics to consider IBS sub-type and to use validated symptom and QoL assessments [41]. In this regard our study only included patients with IBS-D, and assessments were performed using validated IBS-SSS and IBS-QoL instruments.

Another interesting finding in the BDA review was that studies which used multi-strain probiotics seemed to produce better clinical results than single-strain probiotics in terms of global symptoms (14 of 35 studies reported statistically significant benefit and 65% of these used multi-strain probiotics), abdominal pain (8 of 35 studies reported statistically significant benefit and 63% of these used multi-strain probiotics) and QoL (only 2 of 16 studies that measured QoL reported statistically significant benefit, and both of these studies used multi-strain probiotics) [41]. Research teams at our institution (BSMMU) have also seen variable responses dependent upon the bacterial strains used as probiotic. Results of a randomized, double-blind, placebo-controlled trial of the effects of a single strain probiotic, *S. boulardii,* in IBS-D patients were not very promising [32]. However, a follow-up study with a multi-strain probiotic yielded a beneficial outcome which was significant both clinically and statistically [33]. A number of studies have shown a decrease in the microbial diversity of IBS patients compared to healthy controls [42], and specifically in relation to *Bifidobacteria* and *Lactobacilli* spp. [12, 43]. The 14-strain probiotic used in this current study (including 7 *Lactobacilli* and 4 *Bifidobacteria* spp.) may have provided clinical benefits by increasing the microbial diversity in these patients, and may also help explain why single-strain probiotic studies have not been as successful in reducing symptoms in IBS patients.

It is undeniable that the gut microbiota has both direct and indirect effects on the immune system and inflammation [44, 45]. Current evidence suggests that IBS patients have greater mucosal cellularity and other signs of increased inflammatory activity which might contribute to the development of IBS [12, 46]. In this study markers of inflammation were not measured, however future studies would benefit from monitoring inflammation to explore the impact of probiotics on systemic and local inflammatory markers. Overall, and despite the growing interest in this field, our understanding of the role of the gut microbiota in functional GIs such as IBS remains limited [12]. The results of the current trial provide an insight into the benefits that may be obtained using a multi-strain probiotic, but the study was not designed to help elucidate the physiological mechanisms underpinning the observed clinical improvements.

### Study strengths and limitations

The strengths of this clinical trial relate to its design involving a large number of IBS-D patients with strict controls to reduce factors which could influence bias (maintained double-blind by independent personnel) and variation (large number of patients with severe IBS-D in a placebo-controlled study). The vast majority of IBS probiotic trials undertaken so far suffer from small sample sizes, which renders their findings inconclusive. In contrast, this trial appears to be one of the largest published to date in a ‘relatively’ homogeneous group of patients with severe IBS-D. The trial had adequate statistical power to determine statistically/clinical relevant differences between the multi-strain probiotic and placebo in patients with IBS-D. This limits conclusions that can be drawn from findings to similar types of patients treated with the same multi-strain probiotic species.

For a trial of this type there are a number of important limitations that need to be outlined. Firstly, compliance was only checked qualitatively and reinforced at each client visit via verbal questioning and no metrics were maintained. Secondly, while all participants were advised to maintain their usual dietary practices throughout the study, and this was monitored informally at client visits, no nutritional assessments were undertaken to confirm dietary adherence. Thirdly, the reasons for patients withdrawing from the study were not always readily available. Because of this it was decided to perform a per-protocol analysis which presents a potential biased best-case view of the available data since the conclusions only apply to ‘ideal’ patients who are fully adherent to the treatment that they have been given. Another potential limitation of the study is that routine colonoscopy was not performed to rule out the presence of microscopic colitis (MC), as is the case for almost all studies in this therapeutic setting. However, the relatively young age of our cohort (approximate mean age 32 years) with a predominance of males (almost 80%) means that the relative incidence of MC would be low and unlikely to significantly impact on the reported findings.

Finally, the duration of the study (4 months’ treatment plus one month’s follow-up) is consistent with clinical trials in this therapeutic setting, but it is relatively short for a disease which is potentially life-long. Consequently, no conclusions regarding the durability of the response can be made.

## Conclusions

In this large controlled clinical trial well-validated instruments to assess symptom severity (IBS-SSS) and QoL (IBS-QoL) were used in patients with IBS-D. We found that the multi-strain probiotic Bio-Kult® (14 different bacterial strains; 8 billion colony-forming units per day) was safe and superior to placebo in improving GI symptoms over a period of 4 months in patients with IBS-D. Furthermore, symptom improvement was paralleled by statistically significant benefits in all measures of QoL. Finally, it is important to note that the findings only apply to the multi-strain probiotic administered and should not be generalised to other probiotics or IBS patient subtypes.

## Additional file

Additional file 1: The consort checklist. (DOC 254 kb)
